# Inulin and *Lycium barbarum* polysaccharides mitigate the diabetic inflammatory response by modulating bile acid metabolism to enhance regulatory T cell (Treg) activation in a rat model of diabetes

**DOI:** 10.3389/fendo.2025.1705635

**Published:** 2025-12-17

**Authors:** Haixia Lu, Hongyan Luo, Peiling Li, Yan Yang, Shilu Cao, Yanfang Zhang, Hongxia Ma, Qian Zhao, Yali Zheng, Hao Wang

**Affiliations:** 1 Nutritional Department, People’s Hospital of Ningxia Hui Autonomous Region, The Third Clinical College, Ningxia Medical University, Yinchuan, China; 2 Nephrology Department, People’s Hospital of Ningxia Hui Autonomous Region, the Third Clinical College, Ningxia Medical University, Yinchuan, China; 3 General Practice, People’s Hospital of Ningxia Hui Autonomous Region, the Third Clinical College, Ningxia Medical University, Yinchuan, China; 4 Infectious Diseases Department, People’s Hospital of Ningxia Hui Autonomous Region, the Third Clinical College, Ningxia Medical University, Yinchuan, China; 5 Public Health Center, People’s Hospital of Ningxia Hui Autonomous Region, the Third Clinical College, Ningxia Medical University, Yinchuan, China; 6 Department of Pathogenic Biology and Medical Immunology, School of Basic Medical Sciences, Ningxia Medical University, Yinchuan, China

**Keywords:** bile acids, FXR-FGF15-FGFR4 axis, inulin, *Lycium barbarum* polysaccharides, Treg cells, type 2 diabetes mellitus

## Abstract

**Background:**

Type 2 diabetes mellitus (T2DM) is a chronic metabolic disease that poses a serious threat to health. Currently, there are no completely effective treatment options. Modulating intestinal flora and its metabolites may represent a promising new approach for diabetes therapy. Regulating intestinal microbiota and its metabolites through prebiotics, mediated by regulatory T (Treg) cells, could offer a novel strategy to improve the chronic inflammatory state associated with diabetes.

**Objective:**

This study aimed to investigate the improvement of diabetes-related chronic inflammation by examining the effects of inulin (INU) and *Lycium barbarum* polysaccharides (LBP) in diabetic rats. This was achieved by modulating the bile acid metabolites of the intestinal flora through the FXR–FGF15–FGFR4 axis, thereby activating Treg cells *in vivo* and alleviating the inflammatory state associated with diabetes.

**Methods:**

A diabetic rat model was established using a high-fat diet and streptozotocin (STZ) injection. Sprague–Dawley rats were randomly allocated into four groups: type 2 diabetes mellitus (T2DM), T2DM with INU group (T2DM + INU), T2DM with LBP group (T2DM + LBP), and T2DM with INU and LBP group (T2DM + INU + LBP). After 8 weeks of intervention, the rats were euthanized, and relevant pathophysiological and biochemical indicators were analyzed.

**Results:**

INU and LBP treatments significantly decreased the levels of inflammatory cytokines, including MCP-1, IL-18, NF-κB, NLRP3, superoxide dismutase (SOD), and malondialdehyde (MDA). Moreover, these alleviations of the inflammatory state of diabetes were partially attributed to the increased proportion of Treg cells. We found that the abundance of tauro β-muricholic acid (TβMCA) was reduced following INU and LBP treatment, whereas the relative abundances of chenodeoxycholic acid (CDCA), lithocholic acid (LCA), and hyocholic acid (HCA) were all increased compared to those in the untreated group. Mechanically, INU and LBP significantly influenced the negative feedback regulation of the FXR–FGF15–FGFR4 axis via intestinal bile acids, thereby increasing the proportion of Treg cells in the periphery of diabetic rats. Intriguingly, an increase in Treg cells after INU and LBP intervention was notably correlated with the improvement in the inflammatory state of diabetes.

**Conclusions:**

INU and LBP modulate bile acids derived from intestinal flora to improve the chronic inflammatory status of diabetic rats. Specifically, both exert their effectiveness by regulating gut microbial bile acid metabolites through the FXR–FGF15–FGFR4 axis to activate Treg cells. These findings provide an experimental basis for further exploration of the mechanism underlying the effects of this combination in diabetic animal models, which may contribute to clinical therapeutic practice for the control of the disease.

## Graphical Abstract

**Figure:**
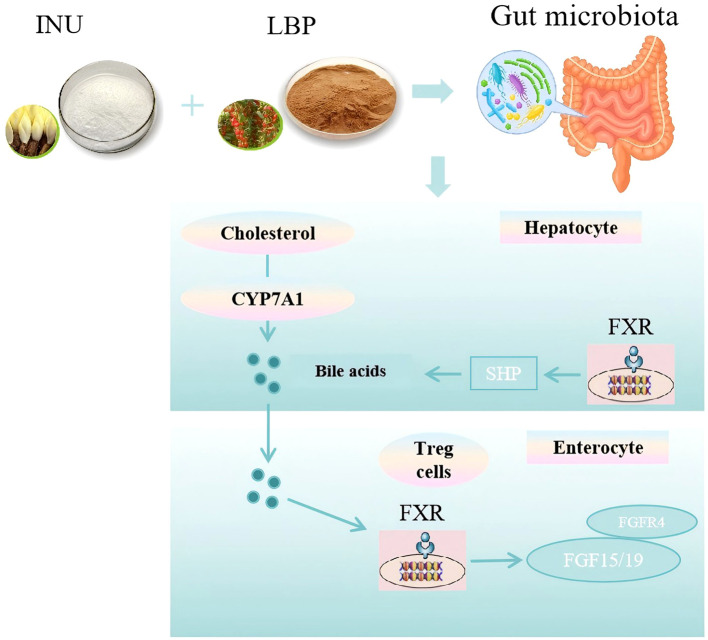

## Introduction

Type 2 diabetes mellitus (T2DM) is a progressive metabolic disease characterized by pancreatic β-cell dysfunction and peripheral insulin resistance (1), leading to impaired glucose metabolism and chronic low-grade inflammation (2). The global prevalence of T2DM is rapidly increasing, contributing significantly to high morbidity and mortality rates. Currently, 537 million people are affected by diabetes, and this number is projected to increase to 783.2 million by 2045 (3). T2DM is now recognized as one of the most challenging endocrine disorders and a major cause of death worldwide. Multiple factors, including host genetics, metabolic alterations, lifestyle, and dietary habits, have been identified as key contributors to the onset and progression of obesity and diabetes. Despite these significant advances, the precise mechanisms underlying the development of T2DM remain unclear. Recent studies have emphasized the pivotal role of the gut in maintaining human health, with gut dysbiosis closely associated with various pathological conditions, particularly T2DM. Consequently, targeting gut microbiota dysbiosis has become a promising therapeutic strategy for T2DM management.

Numerous studies have shown that modulating the composition and metabolites of the gut microbiota can significantly ameliorate T2DM (4). In particular, dietary interventions, especially the use of probiotics and prebiotics, have shown beneficial effects by positively influencing the gut microbiota and its metabolites (5), such as bile acid metabolism, in the management of T2DM. Therefore, innovative dietary strategies for reshaping gut microbiota homeostasis are urgently needed to improve the control and prevention of T2DM.

Inulin (INU) is a dietary prebiotic classified as a fructan-type polysaccharide, primarily sourced from chicory roots. It is resistant to digestion and absorption in the human digestive system but can be fermented by the gut microbiota, ultimately promoting the proliferation of beneficial microorganisms. This fermentation process may help improve insulin resistance and optimize blood lipid regulation in patients with T2DM (6, 7). *Lycium barbarum*, commonly known as wolfberry, is a traditional Chinese herbal medicine widely used because of its health benefits. One of the bioactive properties of *L. barbarum* polysaccharides (LBP), is its ability to play a significant role in immune regulation by activating T cells, B cells, and dendritic cells, as well as reducing blood glucose levels. Additionally, LBP exhibits prebiotic potential by promoting the growth of the gut microbiota (8). In addition, our previous research found that dietary INU and LBP ameliorated diabetes by enhancing the gut barrier, modulating the gut microbiota, and activating gut mucosal TLR2^+^ intraepithelial gammadelta T cells in rats (9).

Multiple studies have demonstrated that intestinal health is closely linked to the immunosuppressive activity of CD4^+^ regulatory T (Treg) cells (10, 11). The activation of colonic Treg cells is mediated by bile acid metabolite produced by intestinal bacteria (12, 13). Regulatory T (Treg) cells characterized, by the expression of the transcription factor forkhead box P3 (FOXP3), are essential for maintaining self-tolerance and immune homeostasis (14, 15). Many studies have shown that the lower expression of anti-inflammatory cytokines in peripheral blood Treg cells plays an important role in the development of diabetes and its associated complications (16, 17). In individuals with T2DM, the percentage of CD4^+^CD25^+^Treg cells in peripheral blood is lower than that in healthy individuals, with an even more pronounced decrease observed in patients with diabetic complications (16). Depletion of Treg cells has been shown to increase blood glucose levels and reduce insulin sensitivity, suggesting that Treg cells are crucial for the development of T2DM. Furthermore, recent studies have shown that host bile acids can induce the production of peripheral Tregs, a function primarily influenced by gut microbes (10, 18).

Previous studies have reported that INU and LBP can improve T2DM by regulating short-chain fatty acids (SCFAs), which may enhance GLP-1 secretion from intestinal L cells (7, 19). Interventions with INU and LBP have been shown to modulate gut microbiota-derived metabolites by increasing intestinal SCFA levels in diabetic models (9). Although SCFAs contribute to glucose metabolism, improvement in diabetes is not solely dependent on the SCFA-GLP-1 pathway.

The gut microbiota also produces a variety of metabolites, including bile acids (BAs), which play important roles in immune regulation, insulin resistance, and energy metabolism (20, 21). Gut microbiota dysbiosis has been associated with abnormal BAs metabolism, particularly in individuals with obesity and T2DM. Importantly, recent evidence indicates that BAs can modulate regulatory T (Treg) cells, which are crucial for controlling inflammation in diabetes. These findings highlight the potential therapeutic role of modulating bile acid metabolism and gut microbiota in promoting Treg cell activity for the management of T2DM and its associated complications.

Consistent with these findings, we hypothesized that INU and LBP ameliorate the inflammatory state of T2DM by activating Treg cells through modulation of the FXR–FGF15–FGFR4 bile acid metabolic pathway. These effects may occur partially independent of SCFAs, highlighting the multifactorial mechanism through which INU and LBP exert anti-diabetic benefits. Therefore, we focused primarily on the role of intestinal-derived bile acids in regulating Treg cells and improving inflammation. This hypothesis was tested using a diabetic rat model to provide experimental evidence for the mechanisms of action of INU and LBP in diabetes.

## Materials and methods

### Establishment of type 2 diabetes

Forty male Sprague–Dawley (SD) rats aged 5 weeks were obtained from the Animal Experiment Center of Ningxia Medical University. The rats were acclimated for 1 week on standard chow in a temperature- and humidity-controlled room. At the beginning of the 7th week, all animals were fed a high-fat diet. After 4 weeks of high-fat diet, streptozotocin (STZ) was used to induce diabetes in rats, as previously described (22). Rats with fasting blood glucose concentrations exceeding 11.1 mmol/L were considered as successfully diabetic. All animal experiments were conducted under the supervision and approval of the Animal Ethics Committee of Ningxia Medical University (No. 2019-033). Male rats were chosen for this study primarily because glucose levels in diabetic male rats are more stable, minimizing interference from the female estrous cycle on the intervention’s efficacy (23).

### Grouping and treatment

After adaptive feeding, SD rats successfully induced with diabetes were ranked in descending order of body weight. Using SPSS 22.0, 40 random numbers were generated and matched with the rats’ weights. The randomized list was then sorted in descending order, with numbers 1–10 assigned to the T2DM group, 11–20 to the T2DM+INU group, 21–30 to the T2DM + LBP group, and 31–40 to the T2DM + INU + LBP group. Daily dosing volumes were adjusted according to real-time body weight (measured with an electronic balance, ± 0.1 g) at 1.5 mL per 100 g body weight. In the T2DM+INU group, inulin was administered orally at 3 g/kg/day; in the T2DM + LBP group, LBP was administered by gavage at 400 mg/kg/day; and in the T2DM + INU + LBP group, both INU (3 g/kg/day) and LBP (400 mg/kg/day) were administered orally. Inulin was provided by Fengning Pingan High-tech Industrial in Hebei, China. LBP was provided by Beryl Wolfberry Co., Ltd. Ningxia, China. After the experiment, the rats were intraperitoneally injected with 3% pentobarbital sodium (50 mg/kg body weight). After confirming anesthesia, the rats were euthanized by dislocation. Serum, liver, spleen, and intestinal samples were collected for further analysis.

### Enzyme-linked immunosorbent assay

The plasma concentrations of SOD (JL, 22893, China), NF-κβ (JL, 21039, China), GLP-1 (JL, 12394, China), INS (JL, 10692, China), NLRP3 (JL, 47550, China), and MDA (Solarbio, BC0025, China) were detected using ELISA kits with the quantitative sandwich enzyme immunoassay technique, according to the manufacturer’s protocols. Briefly, 50 μL of negative and positive control solutions were added separately, and 10 μL of sample and 40 μL of sample dilution were added into wells. Then, 100 μL of horseradish peroxidase (HRP)-labeled detection antigen was added to each well of the negative and positive control and sample wells. The reaction wells were sealed with a plate membrane and incubated at 37 °C for 60 min. After washing each well five times, 50 μL of substrate solution A and 50 μL of substrate solution B were added to each well and incubated at 37 °C for 15 min in the dark. Approximately 50 μL of stop buffer was added to each well, and OD of each well was measured at 450 nm within 15 min.

The concentrations of plasma IL-18 and MCP-1 were detected using the LEGENDplexTM kit (BioLegend, No.740401, USA) according to the manufacturer’s instructions. Briefly, 25 μL of mixed beads were added to each tube. For the standard part, 25 μL of standards at different concentrations were added, and 25 μL of plasma samples were added for detection. Then, 25 μL PE was added to each tube, which was incubated for 2 h at room temperature away from light. Thereafter, 1 mL wash buffer was added to each tube, centrifugation was performed at 300×*g*/min for 5 min, and the supernatant was discarded. Finally, 300 μL wash buffer was added to resuspend the beads, and the samples were measured using a CytoFLEX flow cytometer.

### Western blot analysis

The mixture of loading buffer and extracted proteins from animal intestine samples was boiled for degeneration. Denatured proteins were separated by 4%–12% SDS-PAGE gels as appropriate. After electrophoresis, the proteins were transferred to PVDF (0.45 um pore size) and blocked in the transfer buffer for 1 h. Later primary antibodies against cytochrome P4507A1 (diluted 1:500, Affinity Biosciences DF2612, USA), NR1H4 (diluted 1:500, Affinity Biosciences DF12511, USA), FGF15 (diluted 1:500, Affinity Biosciences DF2651, USA), FGFR4 (diluted 1:500, Protientech 11098-1-AP, USA) and β-actin (diluted 1:3,000, Affinity Biosciences AF7018, USA) were incubated overnight at 4°C. After the membranes were washed with TBS-T buffer for 10 min/round for three rounds, secondary antibodies (Goat Anti-Rabbit or Goat Anti-Mouse, Affinity Biosciences, USA) were applied for an additional 1 h incubation. Chemiluminescent reagents (Affinity Biosciences KF8005, USA) were added prior to the detection of target proteins using a chemiluminescence imager (Odyssey CLx Imager, LICOR, USA).

### Immunohistochemistry analysis

Samples were fixed in 4% formalin, embedded in paraffin, and cut into 4 μm thick sections. After antigen retrieval, blocking buffer was used to block nonspecific sites, and primary antibodies FXR (diluted 1:100, Affinity Biosciences DF12511, USA), CYP7A1 (diluted 1:200, Affinity Biosciences DF2612, USA), FGF15 (diluted 1:200, Affinity Biosciences DF2651, USA), and FGFR4 (diluted 1:50, Protientech 11098-1-AP, USA) were incubated at 4°C overnight. After washing three times with PBS, HRP-IgG (Goat Anti-Rabbit or Goat Anti-Mouse, Affinity Biosciences, USA) was added and incubated for 20 min at room temperature. After three washes with PBS, DAB (ZSbio, PV-9001, China) was added for counterstaining with hematoxylin for 1 min. Graded alcohol was used for dehydration, and the samples were finally observed under a light microscope.

### Quantitative PCR analysis

Total RNA was extracted from rat intestinal tissues using TRIzol reagent (OMEGA Bio-Tech, R6834, USA) according to the manufacturer’s protocol. After identifying the concentration and purity of RNA, cDNA was generated by reverse transcription. Using the Biosystems 7500, the PCR amplification conditions were as follows: 95°C for 30 s and 40 cycles of amplification (95°C for 5 s, 60°C for 34 s). The relative amount of mRNA of each gene was determined using the comparative Ct (ΔCt) method compared with β-actin. The primers used in this study are listed in **Table 1**.

**Table 1:** List of primer used for qRT-PCR.

β-Actin	5’TGTCACCAACTGGGACGATA3’
	3’GGGGTGTTGAAGGTCTCAAA5’
GPBAR1	5’AAGCCTCATCGTCATCGCCAAC3’
	3’TTAGAAAGAAGCAGCCAGCAGGTG5’
FGFR4	5’GTGGCTGCTGTTGGCTTTGTTG3’
	3’GCTCTTGCTGCTCCGAGATTGG5’
FXR	5’AGGATAGAGAGGCAGTGGAGAAGC3’
	3’AGCGTGGTGATGGTTGAATGTCC5’
FGF15	5’GAGGAGGACCAGAACGAACGAAATC3’
	3’CGAGTAGCGAATCAGCCCGTATATC5’
CYP7A1	5’AGGTCTCTGAACTGATCCGTCTACG3’
	3’GAGAATAGCGAGGTGCGTCTTGG5’

### Flow cytometry

Single-cell suspensions from rat spleen tissues and peripheral blood were digested and collected. A 100 μL single-cell suspension was added to a flow tube, 1 μL of CD4-PE (BD, 551397, USA), CD25-PB450 antibodies (BD, 565608, USA), and FITC Anti-FOXP3 antibodies (BD, 11577382, USA) were added to each tube. After gentle mixing and incubation in the dark for 30 min on ice, 1 mL of pre-cooled RPMI-1640 containing 2% fetal bovine serum was added, mixed, and the cells were centrifuged at 350×*g* for 5 min at 4°C. The supernatant was discarded, and the cells were washed again with PBS. The orifice of the flow tube was blotted with filter paper, and 300 μL of RPMI-1640 medium was added to resuscitate the cells. The results were analyzed using flow cytometry.

### Fecal BAs quantification by liquid chromatograph–mass spectrometry

Fecal samples (100 mg) were collected, mixed with 300 μL methanol, oscillated for 1 min, and then centrifuged at 4 °C for 10 min (12,000×*g*) to precipitate the protein. The supernatant was concentrated and dried under vacuum. The solution was dissolved in 100 μL methanol, and the supernatant was analyzed using liquid chromatography–mass spectrometry. UPLC separation was performed using an Acquity UPLC BEH C18 (1.7 μm, 2.1 mm × 100 mm, Waters) column of an Acquity UPLC system (Waters, U.K.). The column temperature was set to 40 °C. The input quantity was 5 μL. The elution gradient of 38 min was as follows: 0 min–4 min, 25% B; 4 min–9 min, 25%–30% B; 9 min–14 min, 30%–36% B; 14 min–18 min, 36%–38% B; 18 min–24 min, 38%–50% B; 24 min–32 min, 50%–75% B; 32 min–35 min, 75%–100% B; 35 min–38 min, 100%–25% B. MS analysis was carried out in negative ion mode using an AB 4000 mass spectrometer (AB, USA) with an ESI source, working in multi-reaction monitoring (MRM) mode. The ion source voltage was 4.5 kV, the source temperature was 500 °C, and the dissolution temperature was 380°C. The collision and curtain gases were set at 6 psi and 30 psi, respectively, and the atomized and auxiliary gases were set at 50 psi.

### Statistical analysis

GraphPad Prism 6.01 was used for the statistical analysis. Measurement data are expressed as mean ± standard deviation. For the metrological data that followed a normal distribution and had homogeneous variance, the t-test was used for analysis between two groups, one-way ANOVA was used for analysis among multiple groups, and the non-parametric test was used for heterogeneity of variance. Correlation analysis was performed using Pearson’s analysis. Statistical significance was set at *P* < 0.05.

## Results

### Dietary INU and LBP ameliorate diabetic inflammation

**Figure 1:**
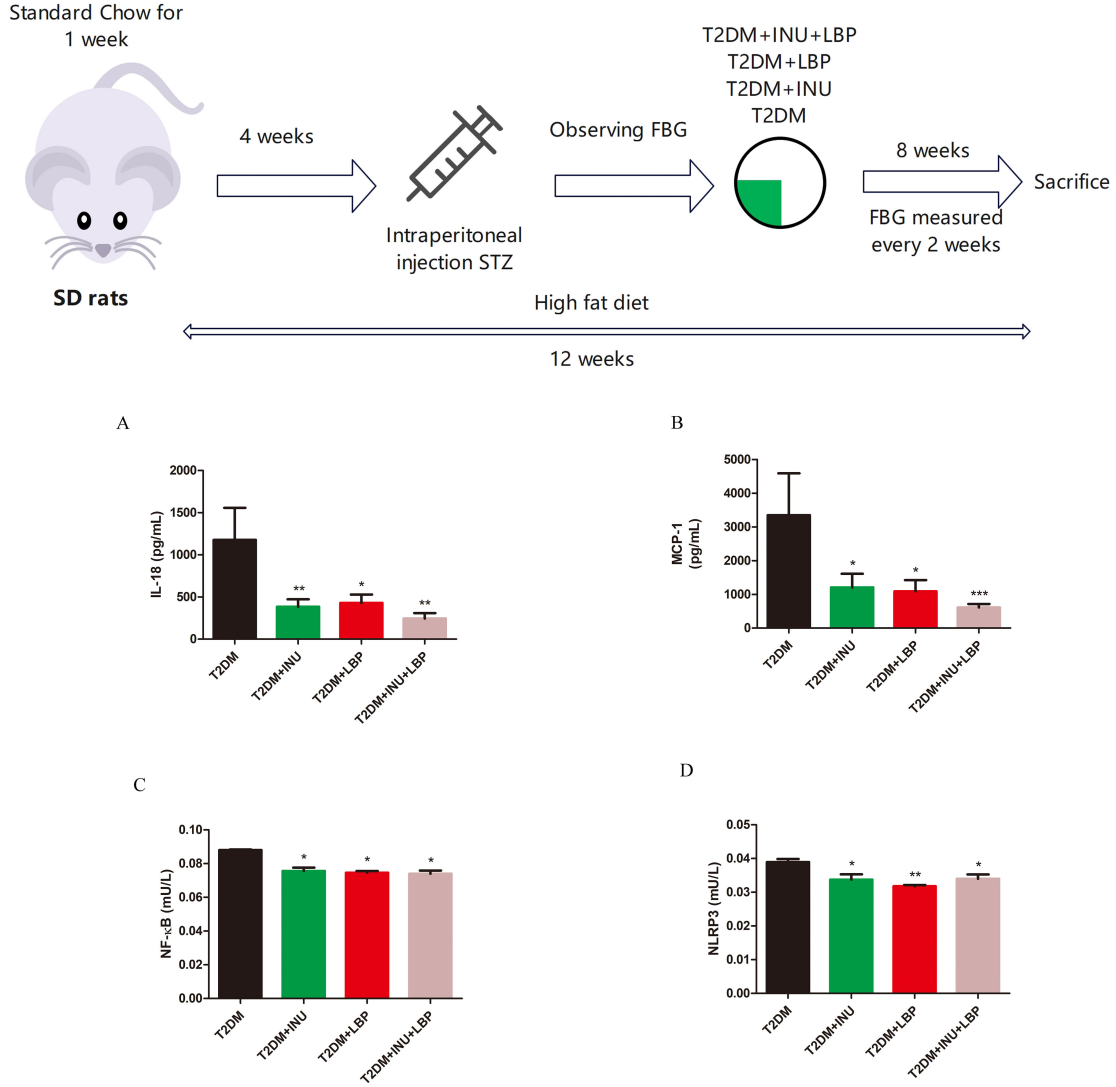
Effects of inulin (INU), *Lycium barbarum* polysaccharide (LBP), and INU + LBP treatment on suppressing inflammation in diabetic rats. Untreated diabetic rats were treated daily with saline. The groups were treated with INU (3 g/kg/d) and/or LBP (400 mg/kg/d) for 8 weeks by oral gavage. Effects of INU and LBP on plasma inflammatory cytokine levels from diverse groups in diabetic rats. Plasma from diverse groups was collected for the detection of **(A)** IL-18, **(B)** MCP-1, **(C)** NF-κB, and **(D)** NLRP3. Data are expressed as mean ± SEM. *P <0.05, **P <0.01, ***P <0.001.

As in our previous studies, SD rats were fed standard chow for 1 week and then induced insulin resistance for 4 weeks. Subsequently, all animals were injected with 35 mg/kg streptozotocin (STZ) to induce a diabetic model. All experimental rats were treated with control saline, INU (3 g/kg/d),0and/or LBP (400 mg/kg/d) for 8 weeks by oral gavage (9). To investigate the effect of INU and LBP on inflammation in diabetes, plasma inflammatory cytokines were detected, and the levels of inflammation pro-inflammatory markers, including IL-18 (INU, *P* = 0.0065; LBP, *P* = 0.0112; INU + LBP, *P* = 0.0015; **Figure 1A**), MCP-1 (INU, *P* = 0.0271; LBP, *P* = 0.0117; INU + LBP, *P <*0.0001; **Figure 1B**), NF-κB (INU, *P* = 0.0130; LBP, *P* = 0.0241; INU + LBP, *P* = 0.0129; **Figure 1C**), and NLPR3 (INU, *P* = 0.0485; LBP, *P* = 0.0020; INU + LBP, *P* = 0.0354; **Figure 1D**) were all significantly decreased following INU, LBP, and INU+LBP intervention compared with the T2DM group.

### Dietary INU and LBP suppressed oxidative stress in T2DM

**Figure 2:**
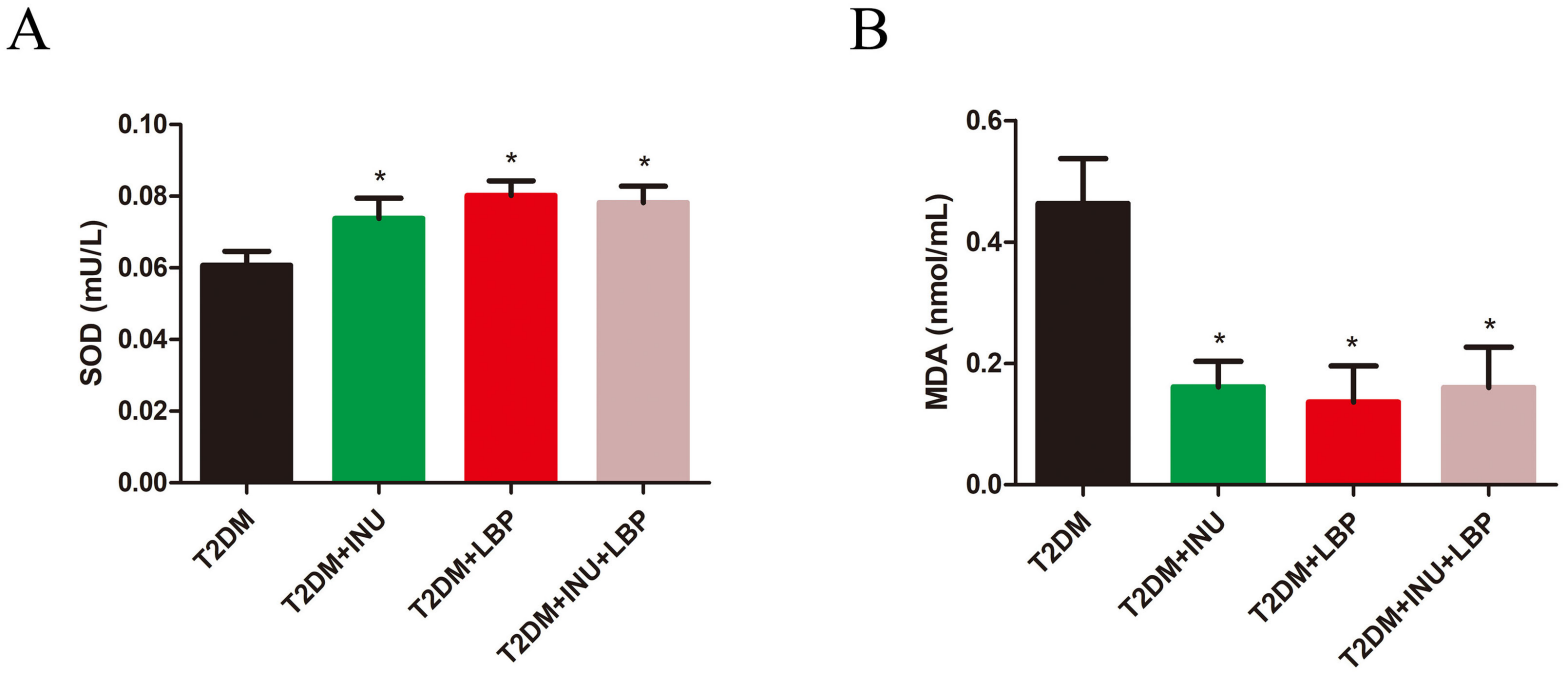
The effects of INU and LBP on the oxidative stress in T2DM. Plasma of diabetic rats from diverse groups were collected respectively for the detection of **(A)** SOD and **(B)** MDA. Data were expressed as mean ± SEM. *P <0.05.

Most studies have shown that oxidative stress is one of the central pathophysiologies of T2DM and is notably increased in hyperglycemic conditions (24, 25). To determine whether INU and LBP treatment could affect oxidative stress in T2DM, the plasma level of superoxide dismutase (SOD) was examined using an ELISA kit, and malondialdehyde (MDA) was analyzed using the oxidation–reduction method. Our results showed that plasma SOD levels were increased in the treated groups compared to the untreated group (INU, *P* = 0.0473; LBP, *P* = 0.0265; INU + LBP, *P* = 0.0453; **Figure 2A**). In contrast, plasma MDA levels (**Figure 2B**) were significantly decreased in the INU (*P* = 0.0239), LBP (*P* = 0.0262), and INU + LBP (*P* = 0.0380) groups.

### Dietary effects of INU and LBP on plasma levels of GLP-1, INS, and FBG

**Figure 3:**
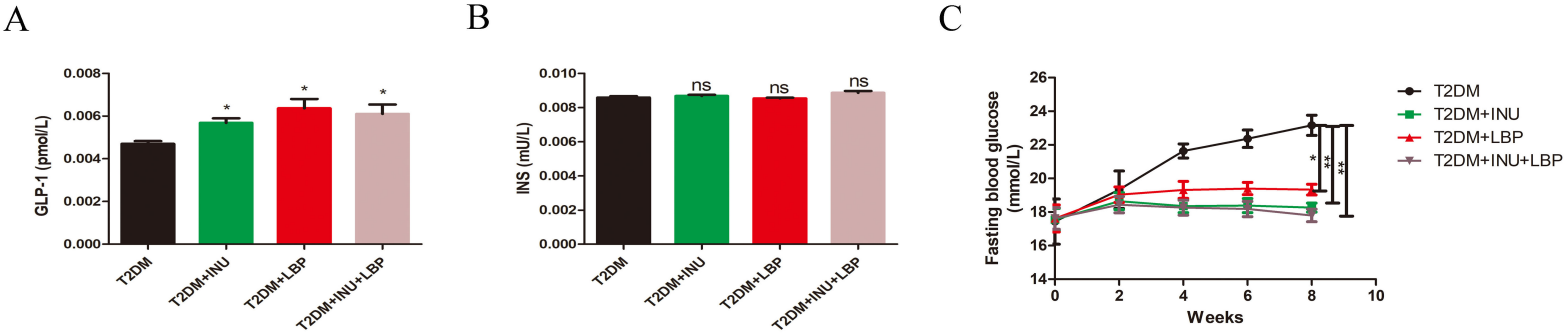
The effects of INU and LBP on plasma GLP-1, insulin, and FBG levels. **(A)** GLP-1. **(B)** INS. **(C)** Fasting blood glucose (FBG). Data were expressed as mean ± SEM. *P <0.05, **P <0.01, ns, no significance.

We next examined the plasma levels of glucagon-like peptide (GLP-1), insulin (INS), and FBG to reflect the effects of INU and LBP treatment on T2DM. Interestingly, GLP-1 (**Figure 3A**) levels were notably increased in the INU group (*P* = 0.0172), LBP group (*P* = 0.0227), and INU + LBP group (*P* = 0.0372) compared with the untreated diabetic group. Insulin levels were measured, and no significant differences were observed among the groups (*P >*0.05, **Figure 3B**). The FBG levels of diabetic rats decreased in the INU group (*P* = 0.0051, **Figure 3C**), LBP group (*P* = 0.0397, **Figure 3C**), and INU + LBP group (*P* = 0.0023, **Figure 3C**).

### Supplementation of INU and LBP modulated Treg cells expression of plasma and spleen in T2DM

**Figure 4:**
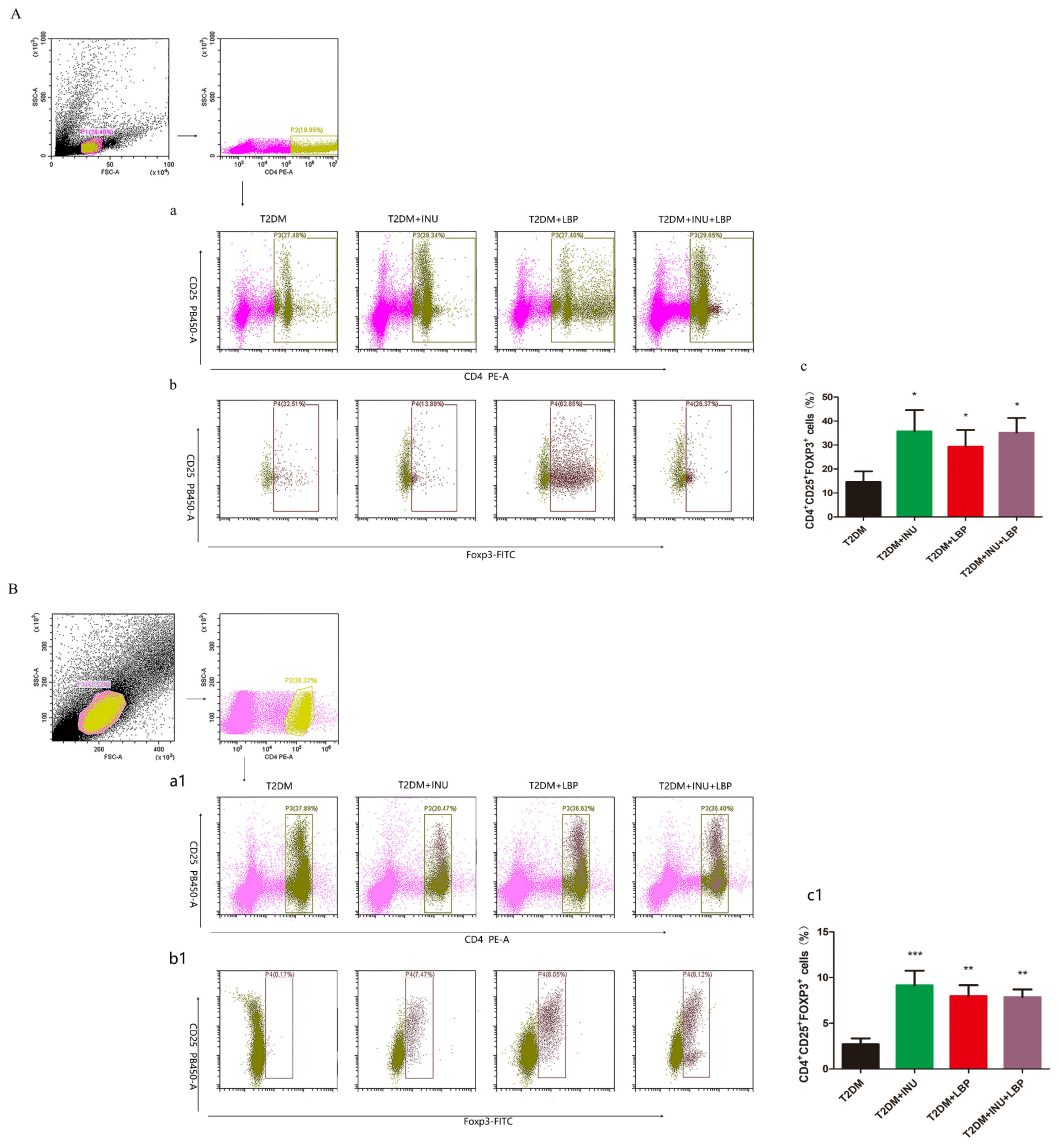
INU and LBP significantly increased the proportion of spleen and plasma CD4^+^CD25^+^FOXP3^+^Treg cells of diabetic rats. CD4^+^CD25^+^FOXP3^+^Treg cells in plasma **(A)** and. spleen **(B)**. Data were expressed as mean ± SEM. *P <0.05, **P <0.01, ***P <0.001.

An increasing amount of evidence has revealed that the progression of T2DM and related metabolic diseases is associated with abnormal immune homeostasis of regulatory T cells (Tregs) (26, 27). Treg cells are crucial for preventing excessive inflammatory responses. To investigate specific immune cell changes among the four groups, we performed flow cytometry to explore Treg expression in the differential intervention groups. As expected, the levels of Treg cells in plasma were significantly decreased in T2DM but increased in the INU group (*P* = 0.0373), LBP group (*P* = 0.046), and particularly in the INU + LBP group (*P* = 0.0103) (**Figures 4A, a–c**). Similarly, the expression levels of Treg cells in the spleen were higher in the INU (*P* = 0.0007), LBP (*P* = 0.0032), and INU + LBP groups (*P* = 0.0038) than in the T2DM group (**Figures 4Ba1, c1**).

### Dietary INU and LBP altered the composition of intestinal flora bile acids in T2DM

**Figure 5:**
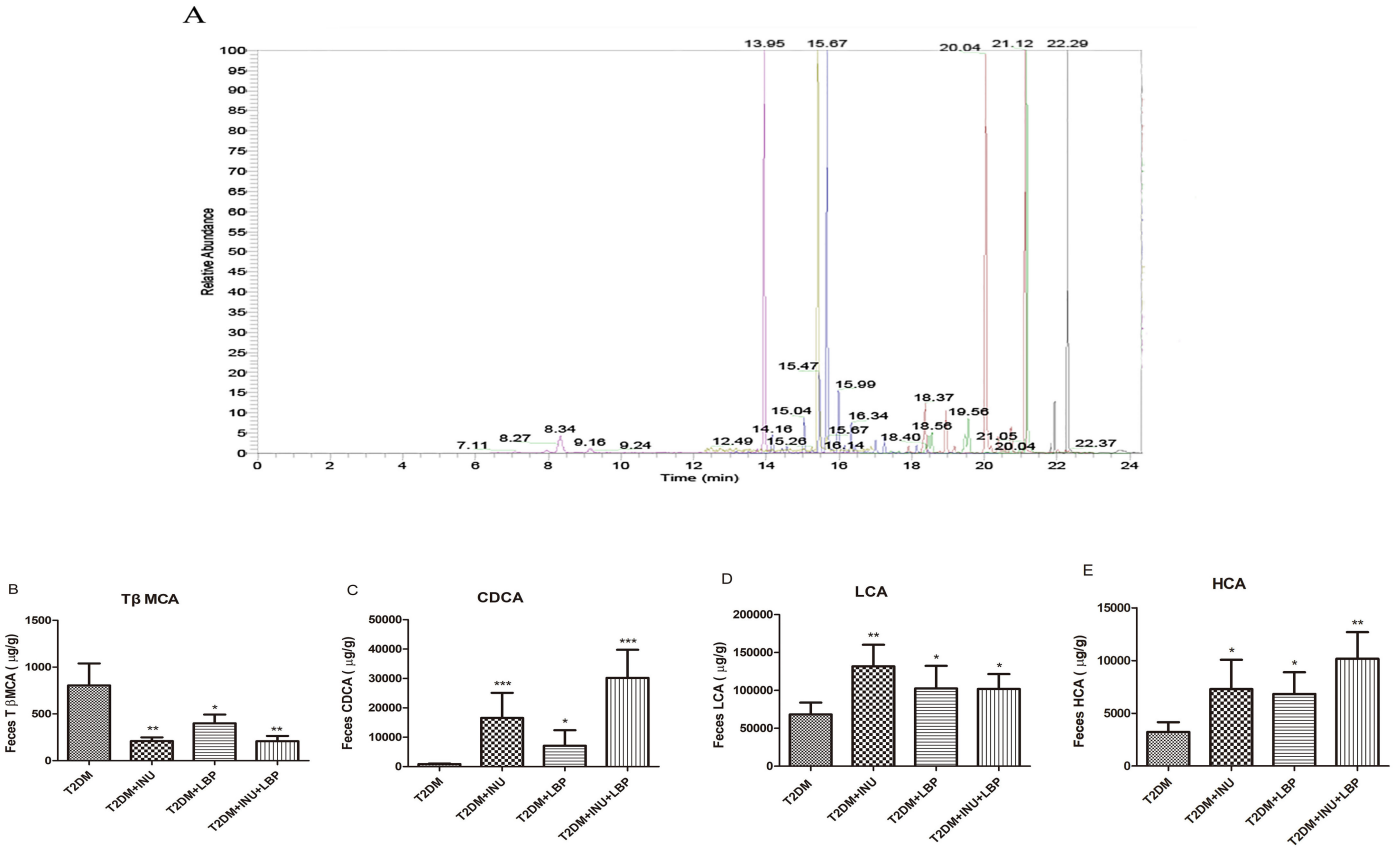
Effects of INU and LBP on the composition of fecal bile acids (BAs) in diabetic rats. Determination of BA components levels (µg/g) by liquid chromatography–mass spectrometry (LC–MS). **(A)** Sample chromatogram of rat feces. The concentrations of several main bile acids in rat stool are plotted in the bar graph **(B–E)**. **(B)** Tauro β-muricholic acid (TβMCA). **(C)** Chenodeoxycholic acid (CDCA). **(D)** Lithocholic acid (LCA). **(E)** Hyocholic acid (HCA). Data were expressed as mean ± SEM. *P <0.05, **P <0.01, ***P <0.001.

Bile acids (BAs), one of the most important metabolites of the gut microbiota, have been suggested to be involved in the progression of chronic metabolic diseases (28, 29). Therefore, we further examined the effects of INU and LBP on BAs using LC–MS (**Figure 5A**). Following INU or LBP intervention, Tauro β-muricholic acid (TβMCA) levels were reduced (INU, *P* = 0.0017, LBP, *P* = 0.0134, INU + LBP, *P* = 0.0020) (**Figure 5B**). Conversely, chenodeoxycholic acid (CDCA) levels were higher in the intervention groups than in the non-intervention group (INU, *P <*0.0001; LBP, *P* = 0.0154; INU + LBP, *P <*0.0001) (**Figure 5C**). Lithocholic acid (LCA) and hyocholic acid (HCA) levels were increased in the INU group (*P* = 0.0098; *P* = 0.0445), LBP group (*P* = 0.0214; *P* = 0.0274), and INU + LBP group (*P* = 0.0228; *P* = 0.0049) compared with the untreated group (**Figures 5D, E**).

### Dietary INU and LBP restored bile acids metabolism homeostasis via FXR–FGF15–FGFR4 pathway in T2DM

**Figure 6:**
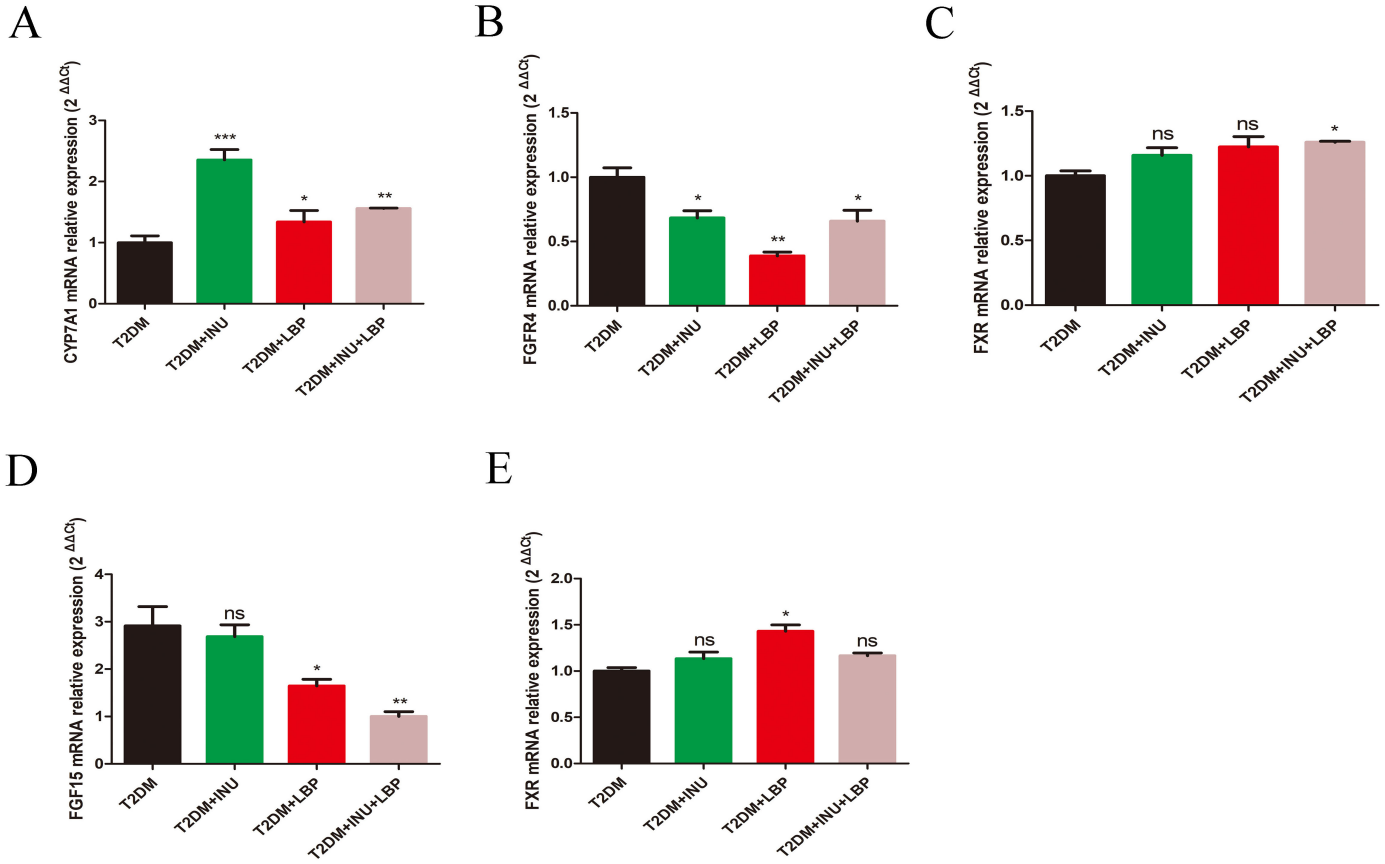
Gene expression associated with bile acid regulation was determined using quantitative real-time PCR. **(A–C)** Semi-quantitative analysis of the relative levels of CYP7A1, FGFR4, and FXR in hepatic tissues. **(D, E)** Semi-quantitative analysis of the relative levels of FGF15 and FXR in the intestine. Data were expressed as mean ± SEM. *P <0.05, **P <0.01, ***P <0.001. ns, no significance. CYP7A1, cholesterol 7a-hydroxylase; FGFR4, fibroblast growth factor receptor 4; FXR, farnesoid X receptor; FGF15, fibroblast growth factor 15.

In recent years, extensive research on bile acid metabolism has revealed that bile acids serve not only as an energy source within the body but also as crucial signaling molecules that regulate sugar and lipid metabolism, energy homeostasis, and inflammation through the activation of distinct signaling pathways in the intestinal-liver circulation system and peripheral organs. The intestinal-liver circulation of bile acids harbors a negative feedback loop involving FXR–FGF15–FGFR4, which is primarily responsible for inhibiting bile acid synthesis. Subsequently, we assessed the hepatic and intestinal expression profiles of FXR–FGF15–FGFR4 axis molecules following INU or LBP treatment in individuals with T2DM. In the liver, the mRNA expression of CYP7A1 was profoundly increased in the INU (*P* = 0.0005), LBP (*P* = 0.0471), and INU + LBP groups (*P* = 0.0073) compared to that in the T2DM group (**Figure 6A**). FGFR4, a specific receptor in the liver for bile acid uptake from the portal, was decreased in the INU (*P* = 0.0266), LBP (*P* = 0.0015) and combined intervention (*P* = 0.0387) groups compared with the untreated group (**Figure 6B**). The mRNA expression of FXR only increased in the combined intervention compared with the T2DM group (INU + LBP, *P* = 0.0128; **Figure 6C**). Additionally, in intestinal sections, the mRNA concentration of FGF15 decreased remarkably in the LBP (*P* = 0.0421) and combined intervention groups (*P* = 0.0013) compared to the T2DM group, but there was no significant change in the INU group (**Figure 6D**). The expression of FXR was only increased in the LBP group (*P* = 0.0212) compared to that in the T2DM group (**Figure 6E**).

**Figure 7:**
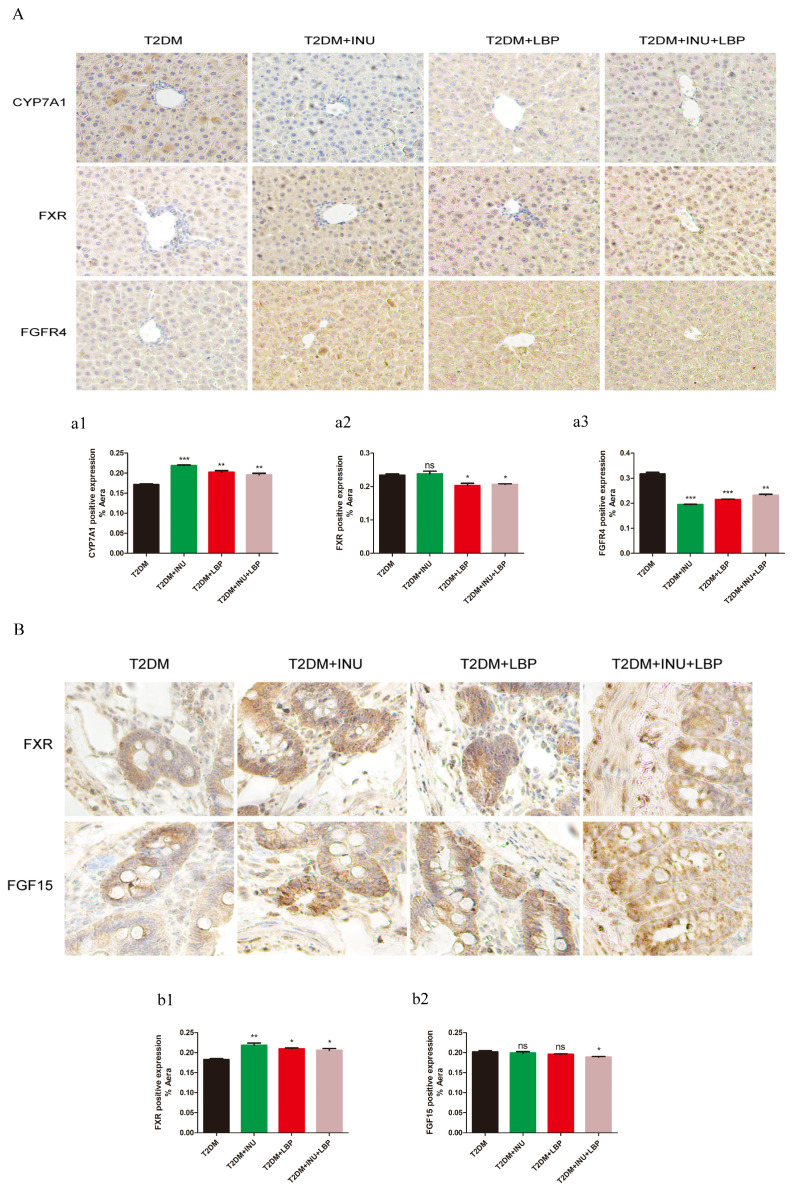
The effects of INU and LBP on bile acid regulation were determined using immunohistochemical methods. **(A)** Immunohistochemical staining of CYP7A1, FXR, and FGFR4 in the liver. **(B)** Immunohistochemical staining for FXR and FGF15 in intestinal tissue. **(a1–a3)**, **(b1, b2)**. Statistical analysis of the proportion of area occupied by positive CYP1A1, FXR, and FGFR4 expression in liver tissue and FXR and FGF15 expression in intestinal tissue. Data were expressed as mean ± SEM. *P <0.05, **P <0.01, ***P <0.001, ns, no significance. Original magnification, ×40.

For further confirmation, the protein expression of related molecules in the liver was detected using immunohistochemical techniques. The expression of the bile acid-limiting enzyme CYP7A1 was increased after INU intervention (*P <*0.0001), LBP intervention (*P* = 0.0017), and INU + LBP intervention (*P <*0.0043) (**Figures 7A, Aa1**). However, the expression of FXR in the INU group was not significantly different from that in the T2DM group, whereas the expression of FXR in the LBP group (*P* = 0.0150) and the combined group decreased (*P* = 0.0166) (**Figures 7A, Aa2**). Histological analysis of liver FGFR4 revealed a notable decrease in the INU (*P <*0.0001), LBP (*P* = 0.0001), and INU + LBP groups (*P* = 0.0042) compared with the untreated group (**Figures 7A, Aa3**).

We further detected the expression of molecules related to bile acid metabolism in the gut using immunohistochemical techniques in intestinal sections. FXR expression was increased with INU (*P* = 0.0036), LBP (*P* = 0.0372), and INU + LBP (*P* = 0.0129) compared to that in T2DM (**Figures 7B, Bb1**). However, the expression of FGF15 showed no significant change after the implementation of INU and LBP intervention alone (*P >*0.05, **Figures 7B, Bb2**). However, FGF15 levels decreased in the INU + LBP group compared with the T2DM group (*P* = 0.0131, **Figures 7B, Bb2**).

**Figure 8:**
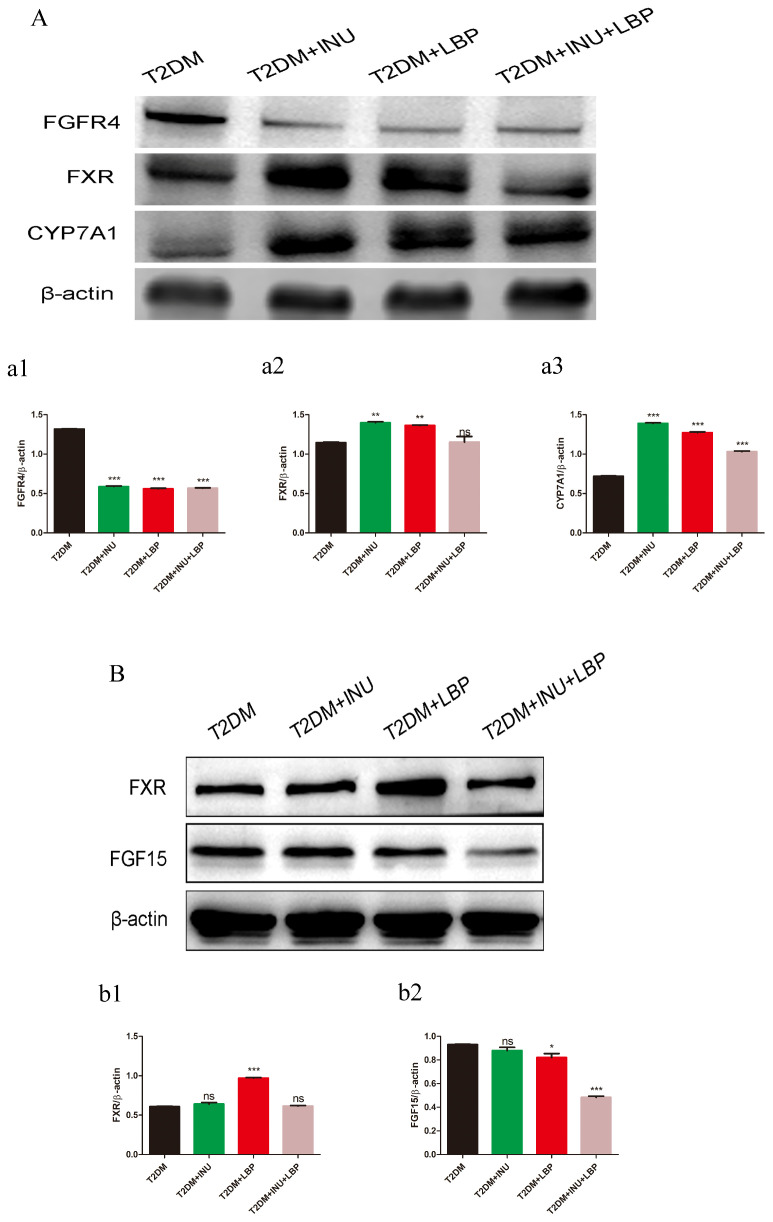
The factors associated with bile acid regulation were examined using Western blotting. **(A)** Western blot analysis of the relative protein expression of FGFR4, FXR, and CYP7A1 in the liver. **(a1–a3)** Semi-quantitative analysis of the relative levels of FGFR4, FXR, and CYP7A1 in the liver by densitometric analysis. **(B)** Western blot analysis of the relative protein expression of FXR and FGF15 in the intestinal tissue. **(b1, b2)** Semi-quantitative analysis of the relative levels of FXR and FGF15 in the intestinal tissue by densitometric analysis. *P <0.05, **P <0.01, ***P <0.001, ns, no significance.

Consistent with the histology results, we performed Western blot analysis to further assess the relative protein expression of bile acids in the liver and intestine sections among the INU, LBP, and combined with LBP groups. The results showed that the relative expression of FGFR4 was significantly decreased after intervention with INU (*P <*0.0001), LBP (*P <*0.0001), and INU + LBP (*P <*0.0001) in the liver (**Figures 8A, Aa1**). The relative expression of FXR was increased in the INU (*P* = 0.0012) and LBP (*P* = 0.0043) groups; however, there was no significant change in the INU + LBP group (**Figures 8A, Aa2**). The relative expression of the rate-limiting enzyme CYP7A1 was notably upregulated in the INU (*P <*0.0001), LBP (*P <*0.0001), and INU + LBP (*P <*0.0001) groups compared to the untreated group (**Figures 8A, Aa3**). Consecutively, in intestinal FXR relative expression was upregulated only in the LBP group (*P <*0.0001) compared to the untreated group in the intestinal sections (**Figures 8B, Bb1**), and the relative expression of FGF15 was decreased in the LBP (*P* = 0.0251) and INU + LBP (*P <*0.0001) intervention groups, whereas there was no significant change in the INU group (**Figures 8B, Bb2**).

## Discussion

In this study, we evaluated the protective effects of INU, LBP, and their combination in diabetic rats by assessing inflammation, plasma oxidative stress, insulin, GLP-1 levels, Treg cells, and bile acid metabolism. Our findings suggest that these interventions may exert their effects in part, through the suppression of the FXR–FGF15–FGFR4 pathway. Additionally, we demonstrated that bile acids derived from the intestinal microbiota may induce Treg cell differentiation. Collectively, these results provide a preclinical foundation for the combined use of INU and LBP in diabetes studies.

Normal control groups with or without dietary treatments were not included for several reasons: (1) previous studies have indicated that INU (30) or LBP (31) have limited effects in healthy animals; (2) our experimental design, based on prior studies (32, 33), focused on diabetes-specific interventions while minimizing animal use; and (3) diabetes modeling relied on fasting blood glucose (FBG) and glycated hemoglobin (GHb) levels (34), independent of control comparisons.

Plasma GLP-1 levels were reduced in untreated diabetic rats but increased following INU, LBP, and combination treatment, suggesting that dietary interventions may enhance glucose homeostasis via GLP-1 secretion. Combined with previous studies indicating that INU and LBP can improve T2DM blood glucose levels (8, 9), this result is considered reliable. However, in our study, the time point selected for GLP-1 measurement (fasting state at the endpoint of the intervention) was validated through preliminary experiments and was suitable for reflecting the steady-state effects of the intervention on the GLP-1 system. Although dynamic monitoring and AUC analyses could provide additional insights, our single-point fasting measurement at the intervention endpoint is consistent with peer-reviewed diabetes studies (35, 36) and sufficiently reflects the steady-state effects. INU and LBP did not significantly enhance insulin secretion, consistent with prior reports (8, 32), indicating that their glucose regulatory effects may be insulin-independent. During our study, we dynamically monitored fasting blood glucose levels and found that both INU and LBP interventions significantly improved fasting glucose levels. However, because insulin levels were not dynamically monitored, we could not confirm whether INU and LBP improved insulin levels in T2DM rats. Further studies are needed to investigate insulin kinetics. It is possible that INU and LBP primarily target chronic inflammation, although the underlying mechanisms have yet to be fully elucidated.

Chronic low-grade inflammation, or “meta-inflammation,” is a hallmark of type 2 diabetes mellitus (T2DM), that disrupts glucose metabolism and insulin signaling in adipocytes, hepatocytes, and muscle cells. Pro-inflammatory cytokines, including IL-18, MCP-1, NLRP3, and NF-κB, play pivotal roles in T2DM pathogenesis (37–39). In our study, INU, LBP, and their combination significantly reduced plasma IL-18, MCP-1, and NLRP3 levels, confirming their anti-inflammatory potential.

IL-18 contributes to insulin resistance and β-cell dysfunction (40, 41) and is upregulated in renal tissues during hyperglycemia (42). INU and LBP interventions effectively reduced IL-18 levels, alleviating diabetes-associated inflammation, suggesting that dietary intake of INU and LBP can alleviate the inflammatory state associated with T2DM in rats. Previous studies have demonstrated that both INU and LBP can elevate interleukin-18 (IL-18) levels in individuals with diabetes mellitus (43, 44). Monocyte chemoattractant protein-1 (MCP-1) is secreted by various cell types, including endothelial cells, monocytes, fibroblasts, vascular smooth muscle cells, and T cells (45). It promotes monocyte chemotaxis and transendothelial migration to inflammation sites by interacting with C–C chemokine receptor 2 (CCR2) on monocytes. Hyperglycemia enhances MCP-1 secretion from kidney cells, leading to tubular macrophage and myofibroblast accumulation, renal fibrosis, and eventual tubular injury (46). Elevated MCP-1 levels in obese and diabetic patients are positively associated with diabetes-related complications (47–49). In this study, INU, LBP, and their combination effectively reduced plasma MCP-1 levels in T2DM rats (**Figure 1**), likely by improving the chronic inflammatory state in diabetic rats. Similar findings have been reported in diabetic models treated with INU and LBP (50, 51).

Nucleotide-binding oligomeric domain-like receptor protein 3 (NLRP3) regulates inflammation and apoptosis (52). Upon activation, it forms the NLRP3 inflammasome, promoting IL-1β and IL-18 release (41, 53). Nuclear factor kappa B (NF-κB) controls the expression of multiple pro-inflammatory genes and plays a key role in in the development of diabetic complications (54, 55). A previous study demonstrated that INU alleviation was closely related to the LPS-TLR4-Mψ-NF-κB-NLRP3 inflammatory pathway (56). Given the critical role of macrophages in T2DM, the anti-inflammatory effects of INU and LBP may involve the inhibition of macrophage activation and polarization. Both treatments markedly reduced plasma NLRP3 and NF-κB levels, suggesting that their protective effects are partly mediated by immune modulation, including Treg cells. Consistent with previous studies, INU or LBP reduced systemic NF-κB and NLRP3 levels under diabetic conditions (57, 58). Further research is required to elucidate the underlying mechanisms of these effects.

Oxidative stress is considered one of a primary factor responsible for the development and progression of T2DM. In this study, dietary intervention with INU or LBP increased serum superoxide dismutase (SOD) levels and decreased malondialdehyde (MDA) levels, which is consistent with previous findings. These results indicate that dietary INU and LBP can alleviate oxidative stress, thereby improving diabetes. Numerous animal and clinical studies have also demonstrated that INU or LBP can reduce oxidative stress by improving SOD activity in diabetes (58, 59). T2DM is a metabolic proinflammatory disorder characterized by chronic hyperglycemia and increased levels of circulating cytokines, suggesting immunological disturbances (60).

Regulatory T cells (Tregs) are a subset of CD4^+^ T cells that maintain immune tolerance and prevent autoimmunity. Their dysfunction is associated with the development and progression of T2DM (61). Clinical studies have reported significantly reduced CD4^+^CD25^+^ Treg levels in T2DM patients, especially those with complications (62, 63). In this study, the proportion of CD4^+^CD25^+^Foxp3^+^ Treg cells in the peripheral blood and spleen of T2DM rats was lowest in the model group but significantly increased after INU and LBP intervention. These findings indicate that INU and LBP exert immunomodulatory effects by promoting Treg cell proliferation. Consistent with previous studies, both prebiotics appeared to regulate immune responses mainly through Treg modulation (64, 65), although the underlying mechanisms warrant further investigation.

Bile acids (BAs) are key signaling molecules that regulate glucose homeostasis, lipid metabolism, and energy expenditure. Synthesized in the liver and maintained via enterohepatic circulation (66, 67), primary BAs are metabolized by gut bacteria into secondary BAs, which play critical roles in host metabolism. Beyond lipid absorption, BAs act as metabolic regulators by activating signaling pathways, and their metabolism is closely linked to the gut microbiota composition, which is often altered in obesity and T2DM (68, 69).

In a previous study, INU and LBP were shown to modulate the gut microbiota by increasing *Bifidobacterium* and *Lactobacillus* abundance. Here, we focused on microbiota-derived BAs and found that INU and LBP interventions increased intestinal BAs levels, particularly those associated with the FXR pathway. Tauro-β-muricholic acid (TβMCA) levels decreased, whereas chenodeoxycholic acid (CDCA), lithocholic acid (LCA), and hyocholic acid (HCA) increased. These findings suggest that INU and LBP may improve metabolic disorders by regulating the gut microbiota-derived bile acids (70, 71).

Bile acids (BAs) interact with the farnesoid X receptor (FXR) and transmembrane G protein-coupled receptor 5 (TGR5) to regulate glycolipid metabolism, energy homeostasis, and signaling in enterohepatic circulation and peripheral organs (72, 73). Natural FXR agonists include CDCA, DCA, CA, and LCA, whereas TβMCA and possibly UDCA act as antagonists (73, 74). FXR antagonists inhibit ileal FXR and FGF15/19 expression, modulating hepatic FXR activity and stabilizing BAs levels (75).

FXR is primarily localized in the liver and small intestine, with bile acids serving as natural ligands. Activated FXR participates in multiple regulatory processes related to glucose tolerance, insulin resistance, lipid homeostasis, and energy metabolism. Gut-derived bile acids activate hepatocellular FXR, which subsequently suppresses SREBP-1c expression through SHP, thereby reducing fatty acid synthesis. By weakening the binding of intestinal FGF15 to the hepatic FGFR4–β-Klotho receptor complex, this pathway alleviates the inhibition of CYP7A1, promotes cholesterol-to-bile acid conversion, and improves glycolipid metabolism.

Previous studies have shown that inulin supplementation alleviates NAFLD by restoring FXR activity through the FXR–FGF15 signaling pathway, increasing hepatic *de novo* bile acid synthesis, and enhancing bile acid excretion (71). Another study reported that LBP upregulates key enzymes, such as CYP7A1 and CYP8B1, activates FXR and SHP, and downregulates the FGFR4–β-Klotho complex, thereby reducing its inhibitory effect on CYP7A1 and enhancing bile acid synthesis in mouse models of hepatic steatosis and hypertriglyceridemia (76).

In our study, we found that INU and LBP activated hepatic FXR through gut-derived bile acids, subsequently reducing FGF15 expression in the intestine and downregulating the hepatic FGFR4 complex. This decreases the inhibition of CYP7A1, enhances bile acid synthesis, and ultimately improves glycolipid metabolism in T2DM rats. These findings suggest that targeting the gut microbiota–BAs axis is a promising strategy for improving glucose metabolism. As prebiotics, INU and LBP may serve as potential interventions for T2DM by modulating gut microbiota-derived bile acid levels, warranting further investigation (77, 78). In this study, FXR was analyzed using three distinct detection methods. However, the immunohistochemical results showed discrepancies with Western blotting (WB) and quantitative polymerase chain reaction (qPCR) data, which necessitated the following explanations. First, qPCR, WB, and IHC measure FXR at different biological levels and exhibit distinct sensitivities. qPCR was used to quantify FXR mRNA levels, which reflect transcriptional activation rather than the final protein output. WB detects *total FXR protein* in homogenized liver tissue and provides high sensitivity and specificity. The upregulation observed in WB is consistent with the mRNA trends from qPCR, suggesting that transcriptional activation is at least partially translated into an increased overall protein abundance. IHC reflects the *spatial localization and regional expression* of FXR in liver sections. Compared with WB, IHC is more influenced by technical factors, including antigen retrieval efficiency, epitope exposure, and local tissue heterogeneity. Drug treatment may induce shifts in FXR subcellular localization (e.g., nuclear–cytoplasmic shuttling) or conformational changes that reduce antibody binding affinity. Additionally, IHC quantification is semi-quantitative and inherently less sensitive than WB, which may lead to apparent downregulation despite increased total protein levels. Second, sample preparation differs substantially across methods; qPCR and WB both use homogenized whole-tissue lysates, providing an integrated measure of average FXR expression across the liver. IHC relies on thin paraffin-embedded sections. Local variations, such as proximity to vessels, bile ducts, or inflammatory foci, may result in non-representative signal intensities. The paraffin embedding and high-temperature deparaffinization steps may partially denature FXR protein or reduce epitope accessibility, decreasing antibody–antigen binding and potentially leading to weaker signals or false-negative results.

Secondary bile acids have immunoregulatory and anti-inflammatory effects on T cell populations (12). For instance, isoLCA promotes Treg cells via VDR, FXR, or the transcription factor REL, whereas isoallo-LCA enhances the differentiation of naïve T cells into regulatory T cells in the gut lamina propria (10). In this study, intestinal-derived bile acid levels increased following INU and LBP interventions, which activated Treg cells through the FXR–FGF15–FGFR4 axis, thereby improving systemic inflammation in diabetes. However, the precise mechanisms by which specific bile acids modulate Treg activation and alleviate diabetic inflammation via this pathway require further investigation.

### Research limitations, transformation potential, and future research direction

A primary limitation of this study is the exclusive use of male rats, which may limit its generalizability. Sex differences in inflammatory markers are well documented, and hormonal fluctuations during the estrous cycle in females can affect insulin sensitivity and metabolic outcomes (79). Future studies will include female and castrated models to assess estrogen’s influence on INU + LBP efficacy, focusing on sex-specific gut microbiota and bile acid metabolism. Furthermore, the absence of dynamic blood glucose and insulin monitoring limits the conclusions to anti-inflammatory effects rather than direct therapeutic efficacy for diabetes. INU, a safe prebiotic, and LBP, a polysaccharide from Barbary wolfberry with a long history of medicinal use, reduced inflammatory factors (IL-8, MCP-1, NF-κB, and NLRP3), regulated microbiota-derived bile acids, and enhanced Treg cells in male diabetic rats.

However, these findings are preliminary and may not fully translate to human T2DM, necessitating further validation through dynamic metabolic testing and stepwise preclinical-to-clinical studies. Multiple studies have confirmed that patients with T2DM exhibit chronic inflammation. Based on these findings, INU and LBP could serve as dietary adjuncts to manage diabetes-related inflammation, particularly in mild cases or high-risk individuals requiring medication. Future research may involve randomized controlled trials in T2DM patients, including low, medium, and high-dose INU/LBP groups alongside placebo controls, with monitoring of inflammatory markers, blood glucose, and insulin resistance (HOMA-IR). Concurrent analysis of gut microbiota and metabolites can clarify the role of the gut microbiota–inflammation regulatory axis in humans. However, challenges such as the hygroscopicity of INU and the poor water solubility of LBP must be addressed to facilitate clinical translation.

## Conclusion

This study demonstrated that dietary INU and LBP improved chronic inflammation in diabetic rats. Mechanistically, these drugs enhance intestinal bacterial-derived bile acids and modulate inflammation by activating the FXR–FGF15–FGFR4 axis and Treg cells. These findings suggest that INU and LBP ameliorate chronic inflammatory status in rats with type 2 diabetes, at least in part, via the gut microbiota–bile acid axis. While providing a scientific basis for their potential use in T2DM, these results are limited to animal models, and their clinical applicability requires validation in additional species models and human trials.

## Data Availability

The raw data supporting the conclusions of this article will be made available by the authors, without undue reservation.
